# Enhanced Expression of NR2B Subunits of NMDA Receptors in the Inherited Glaucomatous DBA/2J Mouse Retina

**DOI:** 10.1155/2013/670254

**Published:** 2013-09-22

**Authors:** Ling-Dan Dong, Jie Chen, Fang Li, Feng Gao, Jihong Wu, Yanying Miao, Zhongfeng Wang

**Affiliations:** ^1^Institutes of Brain Science, Fudan University, 138 Yixueyuan Road, Shanghai 200032, China; ^2^Institute of Neurobiology, Fudan University, 138 Yixueyuan Road, Shanghai 200032, China; ^3^State Key Laboratory of Medical Neurobiology, Fudan University, 138 Yixueyuan Road, Shanghai 200032, China; ^4^Eye & ENT Hospital, Fudan University, 83 Fenyang Road, Shanghai 200031, China

## Abstract

DBA/2J mouse has been used as a model for spontaneous secondary glaucoma. Here, we investigated changes in expression of NMDA receptor (NMDAR) subunits and Cdk5/p35/NMDAR signaling in retinas of DBA/2J mice using Western blot technique. The protein levels of NR1 and NR2A subunits in retinas of DBA/2J mice at all ages (6–12 months) were not different from those in age-matched C57BL/6 mice. In contrast, the protein levels of NR2B subunits, in addition to age-dependent change, significantly increased with elevated intraocular pressure (IOP) in DBA/2J mice at 6 and 9 months as compared with age-matched controls. Moreover, expression of Cdk5, p35 and ratio of p-NR2A^S1232^/NR2A progressively increased with time in both strains, suggestive of activated Cdk5/p35 signaling pathway. However, the changes in these proteins were in the same levels in both strain mice, except a significant increase of p35 proteins at 6 months in DBA/2J mice. Meanwhile, the protein levels of Brn-3a, a retinal ganglion cell (RGC) maker, remarkably decreased at 9–12 months in DBA/2J mice, which was in parallel with the changes of NR2B expression. Our results suggest that elevated IOP-induced increase in expression of NR2B subunits of NMDARs may be involved in RGC degeneration of DBA/2J mice.

## 1. Introduction

Glaucoma, the second leading cause of blindness worldwide, is a neurodegenerative disease characterized by apoptotic death of retinal ganglion cells (RGCs) and progressive visual field loss [1, 2], which is often associated with high intraocular pressure (IOP). Whilst the mechanisms of RGC death in glaucoma still remain a mystery, glutamate excitotoxicity triggered by overactivation of the N-methyl-D-aspartate receptors (NMDARs) may be a potential risk factor for retinal malfunction in glaucoma [3–5]. Indeed, delivery of NMDA channel blockers has been shown to effectively reduce RGC apoptosis in experimental rat glaucoma models [3, 6–8]. Our recent work also showed that cyclin-dependent kinase 5 (Cdk5)/p35-induced elevation of phosphorylated NR2A subunit of NMDARs at S1232 site (p-NR2A^S1232^) may contribute to RGC apoptotic death in experimental glaucomatous rats [9].

DBA/2J mouse is a spontaneous model of glaucomatous neurodegeneration, which develops a progressive form of pigmentary angle-closure glaucoma [10–13]. In these mice, IOPs become elevated by 6 months of age, and continued intraocular hypertension results in progressive RGC degeneration [12–17]. This is similar to what is observed in primary open angle glaucoma, which makes the DBA/2J mice represent a useful model to study mechanisms of RGC death of human glaucoma [1, 11]. Previous studies have demonstrated that NMDAR antagonist memantine treatment significantly increased RGC survival in DBA/2J mice by inhibiting mitochondrial OPA1 and cytochrome c release, decreasing Bax gene expression and increasing Bcl-2 gene expression, suggesting that overactivation of NMDARs in the glaucomatous DBA/2J retina may lead to a distinct mitochondria-mediated RGC death pathway [15, 18]. However, whether expression of NMDARs is changed in DBA/2J retina is largely unknown. The only evidence for this is that no changes in expression of NMDAR subunits were seen in DBA/2J mice by immunohistochemistry in retinal slices [19]. In the present work, we aimed to examine changes in expression of NMDAR subunits and Cdk5/p35/NMDAR signaling in retinas of DBA/2J mice at various ages (3, 6, 9, and 12 months) using Western blot technique. 

## 2. Material and Methods

### 2.1. Animals

Male DBA/2J mice, obtained from The Jackson Laboratory (Bar Harbor, ME, USA), and age-matched C57BL/6 mice, obtained from SLAC Laboratory Animal Co. Ltd (Shanghai, China), were housed on a 12 h light/dark schedule, with standard food and water provided *ad libitum*. All experimental procedures described here were carried out in accordance with the National Institutes of Health (NIH) guidelines for the Care and Use of Laboratory Animals and the guidelines of Fudan University on the ethical use of animals. During this study, all possible efforts were made to minimize the number of animals used and their suffering.

### 2.2. IOP Measurement

IOPs of both eyes in DBA/2J mice and age-matched C57BL/6J mice were measured using a handheld digital tonometer (TonoLab, TioLat, Finland) under general and local anesthesia as described previously [9, 20]. The average value of five consecutive measurements with a deviation of less than 5% was accepted. All measurements were performed in the morning to avoid possible circadian difference. 

### 2.3. Western Blot Analysis

Western blot analysis was conducted as previously described with some modifications [9, 20]. DBA/2J mice or age-matched C57BL/6 mice in different ages (3, 6, 9, and 12 months) were deeply anesthetized with 25% urethane (1.25 g/kg, i.p.). The retinas were removed quickly and snap frozen in liquid nitrogen and then stored at −80°C for further use. Retinas were homogenized in RIPA lysis buffer (50 mM Tris-Cl, 150 mM NaCl, 1% Triton X-100, 0.1% aprotinin, 1 mM phenylmethylsulfonyl fluoride, 1 mM sodium orthovanadate, and 25 mM sodium fluoride, pH 7.4), supplemented with protease and phosphatase inhibitor cocktail (Roche, Mannheim, Germany). The concentration of total proteins was measured using a standard bicinchoninic acid (BCA) assay kit (Pierce Biotechnology, IL, USA). The extracted protein samples (20 *μ*g, 15 *μ*L in volume) were resolved by 8% or 15% SDS-PAGE gel and electroblotted onto PVDF membranes (Immobilon-P, Millipore, Billerica, MA, USA) using Mini-PROTEAN 3 Electrophoresis System and Mini Trans-Blot Electrophoretic Transfer System (Bio-Rad, Hercules, CA, USA). After blocking in 5% nonfat milk at room temperature for 1 h, the membranes were incubated overnight at 4°C with primary antibodies. The primary antibodies used in the present work include anti-NR1 (#05432, 1 : 500, Millipore, Billerica, MA, USA), anti-p-NR2A^S1232^ (#2056, 1 : 500, Tocris Bioscience, MO, USA), anti-NR2A (#320600, 1 : 500, Invitrogen, Carlsbad, CA, USA), anti-Brn-3a (sc-8429, 1 : 1000, Santa Cluz, Biotechnology, CA, USA), anti-Cdk5 (#20502, clone DC17, 1 : 1000, Millipore), anti-NR2B (#06600, 1 : 500, Millipore), anti-p35/p25 (sc-820, 1 : 1000; Santa Cruz Biotechnology), *β*-actin antibody (A5316, 1 : 2000, Sigma, Saint Louis, MO, USA), and anti-GAPDH (1 : 1000, Cell Signal Technology, MA, USA). After washing in Tris-buffered saline-Tween 20, the membranes were incubated with horseradish-peroxidase-(HRP-) conjugated donkey anti-mouse or donkey anti-rabbit secondary antibody (Thermo Scientific, Rockford, IL, USA) at a 1 : 2500 dilution for 1 h at room temperature. The blots were then incubated with chemifluorescent reagent ECL (Thermo Scientific, Rockford, IL, USA) and exposed to X-ray film in the dark. The experiments were performed in triplicate, and the protein bands were quantitatively analyzed with NIH Image J Analysis software.

### 2.4. Statistical Analysis

All data are presented as mean ± S.E.M. Statistical analysis was performed by using the Graphpad Prism software (version 5.0; Graphpad Software, San Diego, CA, USA). A one-way or two-way analysis of variance (ANOVA) with nonparametric test (Kruskal-Wallis test), Bonferroni's post hoc test (multiple comparisons), and Mann-Whitney test (comparisons between two groups) was used as appropriate. A value of *P* < 0.05 was considered significant.

## 3. Results

### 3.1. Changes in Expression of NMDAR Subunits in DBA/2J Mice at Various Ages

**Figure 1:**
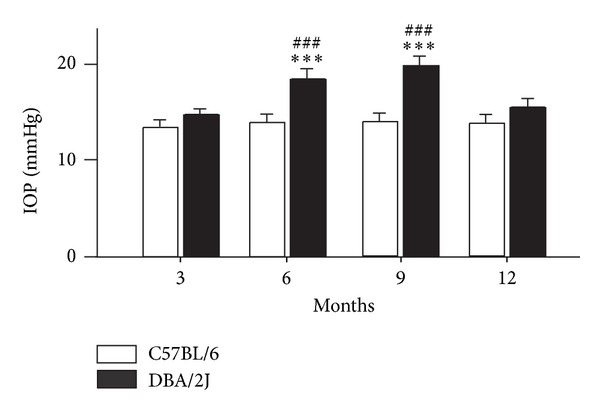
*Changes of IOPs in C57BL/6 and DBA/2J mice at various ages.* Bar chart showing changes of the average IOPs in C57BL/6 and DBA/2J mice at ages of 3 (*n* = 24), 6 (*n* = 18), 9 (*n* = 12) and 12 (*n* = 6) months. All data are presented as mean ± S.E.M. ***P < 0.001 versus 3-month-old C57BL/6 mice; ^###^ *P* < 0.001 versus age-matched C57BL/6 mice.

We first monitored changes in IOPs of DBA/2J mice with time. As shown in Figure 1, the average IOP of DBA/2J mice was 14.8 ± 0.5 mm Hg (*n* = 24) at age of 3 months, which was comparable to that of age-matched C57BL/6 control mice (13.6 ± 0.6 mm Hg) (*n* = 24). The average IOP of control mice kept this level at ages of 6, 9, and 12 months, while that of DBA/2J mice significantly increased to 18.5 ± 0.9 mm Hg (*n* = 18, *P* < 0.001) and 19.9 ± 1.0 mm Hg (*n* = 12, *P* < 0.001) at ages of 6 and 9 months, respectively, and then declined to 15.6 ± 0.8 mm Hg (*n* = 6, *P* > 0.05) at age of 12 months (Figure 1). In the present study, all of the DBA/2J mice showed progressive elevated IOPs. Therefore, our data included all animals.

**Figure 2:**
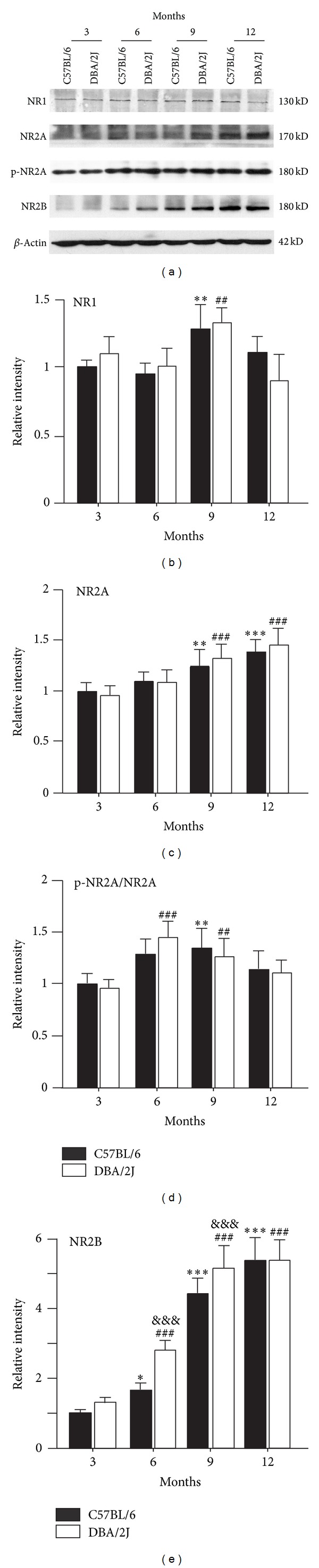
*Changes in expression of NMDA receptor subunits of DBA/2J mice at various ages.* (a) Representative immunoblots showing the changes of NR1, NR2A, p-NR2A^S1232^, NR2B levels in DBA/2J and C57BL/6 mice at ages of 3, 6, 9, and 12 months. (b)–(e) Bar chart showing the average densitometric quantification of immunoreactive bands of NR1 (b), NR2A (c), p-NR2A^S1232^/NR2A (d), and NR2B (e) in DBA/2J and C57BL/6 mice at ages of 3, 6, 9, and 12 months, respectively. *P < 0.05, **P < 0.01 and ***P < 0.001 versus 3-month-old C57BL/6 mice; ^##^ *P* < 0.01 and ^###^ *P* < 0.001 versus 3-month-old DBA/2J; ^&&&^ *P* < 0.001 versus age-matched C57BL/6 mice.

Expression of NR1 subunit of NMDARs was unchanged at the age of 6 months both in DBA/2J and C57BL/6 mice (101.0 ± 13.2% and 95.0 ± 8.1% of that at age of 3 months (control), *n* = 6, *P* > 0.05) (Figures 2(a) and 2(b)). While expression of this protein increased to 133.4 ± 11.2% of control at age of 9 months in DBA/2J mice (*n* = 6, *P* < 0.01), following by a decline to 90.1 ± 19.2% of control at age of 12 months, it was not different from that of age-matched C57BL/6 mice (128.2 ± 17.1% of control, *P* < 0.01 for 9 months; 110.2 ± 12.1% of control, *P* > 0.05 for 12 months, resp.) (Figures 2(a) and 2(b)). Expression of NR2A subunit was elevated at ages of 9 and 12 months in DBA/2J mice (135.3 ± 14.1% and 148.2 ± 17.2% of control, resp., *n* = 6, all *P* < 0.001), which was comparable to age-matched C57BL/6 mice (125.3 ± 16.1% (*n* = 6, *P* < 0.01) and 140.0 ± 12.1% of control (*n* = 6, *P* < 0.001), resp.) (Figures 2(a) and 2(c)). Meanwhile, the ratio of p-NR2A^S1232^/NR2A showed a remarkable increase at ages of 6 and 9 months in DBA/2J mice (145.4 ± 16.0% (*n* = 6, *P* < 0.001) and 128.0 ± 17.0% of control (*n* = 6, *P* < 0.001), resp.), and at the age of 6 months in age-matched C57BL/6 mice (134.5 ± 19.0% of control, *n* = 6, *P* < 0.01) (Figures 2(a) and 2(d)). However, the ratio of p-NR2A^S1232^/NR2A did not show significant difference between DBA/2J and age-matched C57BL/6 mice at all ages. Expression of NR2B subunit was quite different (Figure 2(a)). The average level of NR2B proteins increased to 166.0 ± 19.1% (*n* = 6, *P* < 0.05), 446.3 ± 15.0% (*n* = 6, *P* < 0.001), and 541.4 ± 19.3% of control (*n* = 6, *P* < 0.001) in C57BL/6 mice at ages of 6, 9, and 12 months. Expression of NR2B proteins in DBA/2J mice showed a dramatic increase, with average protein levels being 287.0 ± 29.4% (*n* = 6, *P* < 0.001 versus control and *P* < 0.001 versus age-matched C57BL/6 mice), 517.3 ± 62.0% (*n* = 6, *P* < 0.001 versus control and *P* < 0.001 versus age-matched C57BL/6 mice) and 540.4 ± 58.1% of control (*n* = 6, *P* < 0.001) at ages of 6, 9, and 12 months, respectively (Figures 2(a) and 2(e)). 

### 3.2. Changes in Expression of Cdk5 and p35 in DBA/2J Mice at Various Ages

**Figure 3:**
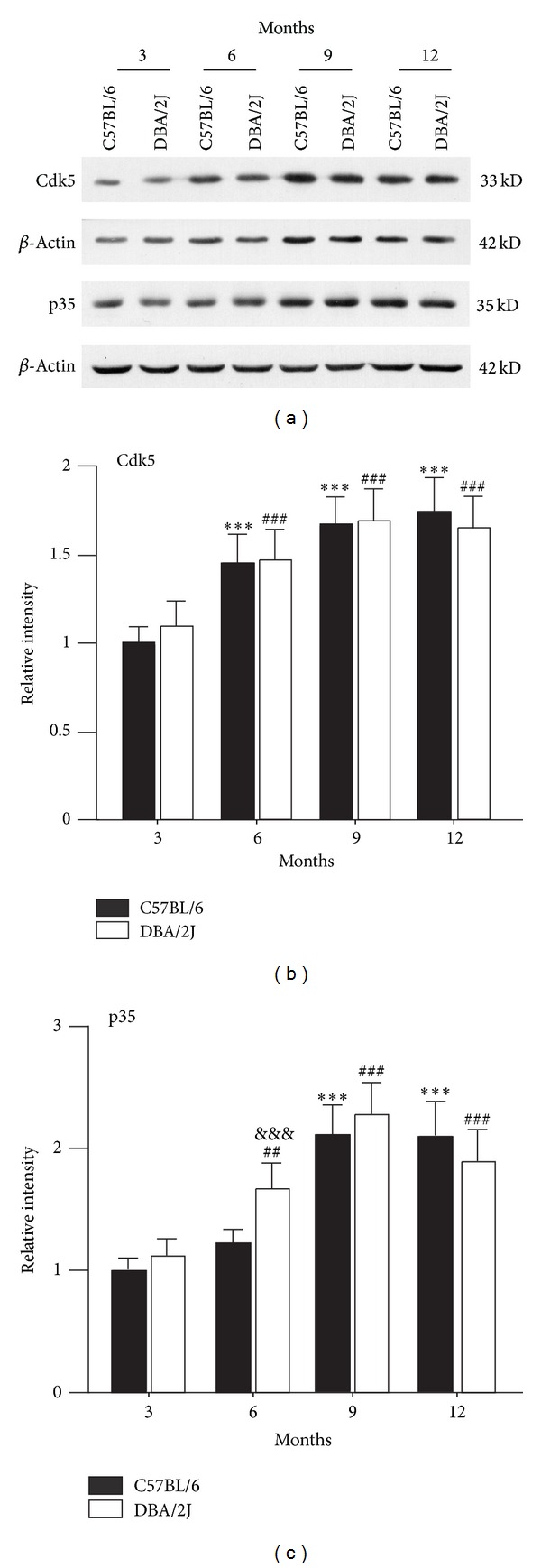
*Changes in expression of Cdk5, p35 of DBA/2J mice at various ages.* (a) Representative immunoblots showing the changes of Cdk5 and p35 levels in DBA/2J and C57BL/6 mice at ages of 3, 6, 9, and 12 months. ((b), (c)) Bar chart showing the average densitometric quantification of immunoreactive bands of Cdk5 (b) and p35 (c) in DBA/2J and C57BL/6 mice at ages of 3, 6, 9, and 12 months, respectively. ***P < 0.001 versus 3-month-old C57BL/6 mice; ^##^ *P* < 0.01 and ^###^ *P* < 0.001 versus 3-month-old DBA/2J; ^&&&^ *P* < 0.001 versus age-matched C57BL/6 mice.

Since the expression of p-NR2A^S1232^ was at high level (Figure 2), we examined changes of its activator Cdk5 and coactivator p35/p25 [9]. As shown in Figure 3(a), the protein level of Cdk5 progressively increased with time in DBA/2J mice, with the average protein density being 148.1 ± 17.0% (*n* = 6, *P* < 0.001), 169.7 ± 18.1% (*n* = 6, *P* < 0.001), and 165.8 ± 18.0% of control (*n* = 6, *P* < 0.001) at ages of 6, 9, and 12 months, respectively (Figure 3(b)). However, there was no significant difference between DBA/2J mice and age-matched C57BL/6 mice at all ages (Figures 3(a) and 3(b)). Similarly, the p35 protein level gradually increased to 168.7 ± 21.1% (*n* = 6, *P* < 0.01), 229.7 ± 26.3% of control (*n* = 6, *P* < 0.001) in DBA/2J mice at ages of 6 and 9 months, and then slightly declined to 191.3 ± 25.1% of control (*n* = 6, *P* < 0.001) at the age of 12 months (Figures 3(a) and 3(c)). Except at age of 6 months (*n* = 6, *P* < 0.001 versus age-matched C57BL/6 mice), the p35 protein levels at all other ages were comparable to those of age-matched C57BL/6 mice (Figures 3(a) and 3(c)). At the same time, p25 protein, a truncated form of p35, was not detected both in DBA/2J and C57BL/6 mice. 

### 3.3. Changes in Expression of Brn-3a in DBA/2J Mice at Various Ages

**Figure 4:**
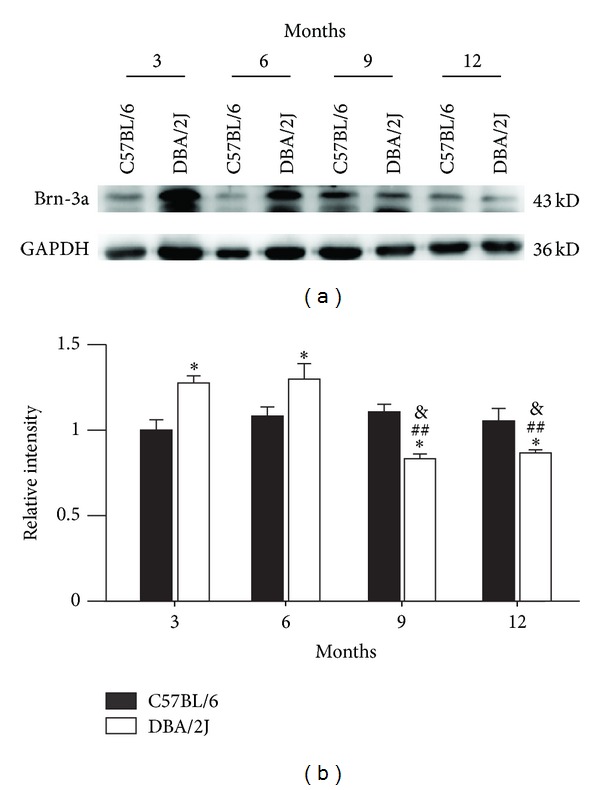
*Changes in expression of Brn-3a of DBA/2J mice at various ages.* (a) Representative immunoblots showing the changes of Brn-3a levels in DBA/2J and C57BL/6 mice at ages of 3, 6, 9, and 12 months. (b) Bar chart showing the average densitometric quantification of immunoreactive bands of Brn-3a in DBA/2J and C57BL/6 mice at ages of 3, 6, 9, and 12 months, respectively. *P < 0.05 versus 3-month-old C57BL/6 mice; ^##^ *P* < 0.01 versus 3-month-old DBA/2J mice; ^&^ *P* < 0.05 versus age-matched C57BL/6 mice.

We finally examined changes in expression of Brn-3a, a RGC marker, to evaluate RGC damage. Figure 4(a) shows representative Western blot results. The protein level of Brn-3a in DBA/2J mice was higher than that of control mice at ages of 3 and 6 months (127.6 ± 4.8% and 129.9 ± 5.4% of control, *n* = 4, all *P* < 0.05), whereas it decreased to 83.3 ± 2.7% (*n* = 4, *P* < 0.05 versus control and age-matched C57BL/6 mice; *P* < 0.01 versus DBA/2J mice at age of 3 months) and 86.7 ± 1.8% of control (*n* = 4, *P* < 0.05 versus control and age-matched C57BL/6 mice; *P* < 0.01 versus DBA/2J mice at age of 3 months) at ages of 9 and 12 months (Figure 4(b)). 

## 4. Discussion

In the present study, we found that expression of NR2B subunits of NMDARs in retinas of the DBA/2J mice was gradually enhanced with time. Progressive elevated IOP-induced increase in NR2B expression may be associated with RGC degeneration in this glaucomatous model. 

Glutamate excitotoxicity has been implicated in glaucomatous RGC death, which is primarily mediated by NMDARs [3, 21]. As a spontaneous glaucomatous model, overactivation of NMDARs by glutamate may be also involved in RGC degeneration of DBA/2J mice. This was supported by the experimental results. First, it was reported that vitreal glutamate content measured with HPLC in DBA/2J mice was higher than that of age-matched controls, and glutamate transporters GLAST and GLT-1v expression in DBA/2J mice showed a decrease by Western blotting [19]. Secondly, blocking NMDARs by memantine inhibited RGC apoptosis and increased RGC survival in DBA/2J mice [15, 18]. It should be noted that time-dependent loss of RGCs in DBA/2J mice started at 6 months of age [16], in parallel with progressive elevation of IOPs and changes of glutamate transporters and glutamate concentration [19]. 

Previous work has demonstrated that expression of both NMDAR and AMPA receptor (AMPAR) subunits did not show age-dependent change in the retinas of DBA/2J mice by immunohistochemistry [19]. Consistent with this, our results revealed that there was no significant change in expression of NR1 subunits in the retina of DBA/2J mice as compared with age-matched C57BL/6 mice by Western blot analysis. In human glaucomatous eyes, however, NR1 levels showed a decrease [22]. Even though there was no difference between DBA/2J mice and age-matched C57BL/6 mice in the expression of NR2A subunits at all ages, age-dependent increase in NR2A expression was observed at 9 and 12 months in both DBA/2J and C57BL/6 mice (Figure 2(c)). A major finding in this work is that, in addition to age-dependent change, expression of NR2B subunits significantly increased accompanyied with elevated IOPs in DBA/2J mice (Figure 2(e)). It was recently reported that NMDARs located at the synapse stimulate cell survival pathways, while extrasynaptic receptors signal for cell death [23–26]. NR2B subunits are commonly associated with extrasynaptic locations at the synapse, thus involving in neurologic diseases and some neurodegenerative disorders [24–30]. In the retina, it was reported that NR2A and NR2B subunits were expressed predominantly synaptically and perisynaptically respectively [31]. Therefore, we deduced that excessive glutamate may stimulate the overexpressed extrasynaptic NR2B subunits in DBA/2J mice, thus triggering cell death signals. Consistently, Brn-3a expression was decreased following the changes of NR2B expression (Figure 4), indicating a correlation between overexpression of NR2B and RGC damage. Although Brn-3a expression was highly related to increase of NR2B expression, it is noteworthy that overactivation of NMDARs is not a sole factor for RGC degeneration in DBA/2J mice. Moreover, Brn-3a expression in DBA/2J mice was higher than control at ages of 3 and 6 months. It is possible that as a transcript factor, Brn-3a protein levels could not accurately reflect the number of RGCs, which is worthwhile to be further explored.

Our previous work found that Cdk5/p35 signaling pathway was activated in a rat experimental glaucoma model, and the activated Cdk5/p35 signaling in turn induced an elevation of p-NR2A^S1232^ expression, which contributed to rat RGC apoptotic death [9]. Indeed, we found that the expression of Cdk5, p35 and the ratio of p-NR2A^S1232^/NR2A progressively increased with time in retinas of DBA/2J mice, suggestive of activated Cdk5/p35 signaling pathway. However, the changes in these protein levels were comparable to those in age-matched C57BL/6 mice, except a significant increase in p35 expression of DBA/2J mice at age of 6 months. These results suggest that progressive moderate elevation of IOP in DBA/2J mice is unlikely a primary factor for activation of Cdk5/p35/NMDAR signaling pathway. Aging plays an important role in activating this signaling pathway. On the other hand, activated Cdk5/p35 signaling pathway may modulate NR2B subunits and increase their expression in DBA/2J mice since it was reported that Cdk5 may indirectly regulate NR2B [32]. 

In conclusion, our results suggest that NMDARs may be involved in RGC degeneration of DBA/2J mice through two pathways: IOP elevation-induced increase in expression of NR2B subunits and age-dependent activation of Cdk5/p35/NMDAR signaling pathway.
